# Separating Myths From Facts About Bread and Health

**DOI:** 10.1111/nbu.70038

**Published:** 2025-11-28

**Authors:** Peter R. Shewry, Alison Lovegrove, Edward J. M. Joy, Lucia Segovia de la Revilla, Gary Frost, Fred Brouns

**Affiliations:** ^1^ Rothamsted Research Harpenden Hertfordshire UK; ^2^ London School of Hygiene & Tropical Medicine London UK; ^3^ Section of Nutrition, Department of Diabetes, Endocrinology and Metabolism, Faculty of Medicine Imperial College London London UK; ^4^ Department of Human Biology, Faculty of Health, Medicine and Life Sciences, School for Nutrition and Translational Research in Metabolism (NUTRIM) Maastricht University Maastricht the Netherlands

**Keywords:** adverse effects, bread, Chorleywood bread process, health benefits, UPFs, wheat

## Abstract

White bread remains a staple food in many countries and global consumption continues to increase. However, there is an increasingly contentious debate, carried out particularly in social media and the popular press, about the adverse effects on health of factory‐produced sliced white bread as opposed to the whole grain breads made with traditional processes, with the classification of factory‐produced sliced bread as ‘ultra‐processed’ adding to these concerns. We examine the scientific basis for this debate and conclude that, despite the loss of bran and germ components during milling and the use of additives, factory‐produced white bread is not intrinsically unhealthy. We therefore conclude that while wholegrain bread is generally recommended as a healthier choice, both white and wholegrain breads have a place in a healthy diet when consumed in moderation and as part of an overall nutrient‐rich eating pattern.

## 1 Introduction

Wheat accounts for about 20% of the total calories consumed globally and production continues to increase, by almost 100 million tonnes over the past decade to a current global level of about 800 million tonnes per annum (FAOSTAT 2025). This increased production reflects higher demand, particularly in countries undergoing rapid urbanisation and industrialisation, which include low and middle‐income countries in Asia and Africa. Most of these countries are net importers of wheat, accounting for most of the 25% of the crop that is globally traded (Erenstein et al. 2023).

However, while the global consumption of wheat is increasing an opposite trend is observed in some traditional wheat‐consuming countries, notable in North America and parts of Europe, where the contribution of wheat to the diet is static or declining (Erenstein et al. 2023). For example, the consumption of bread in the UK was over 950g per person, per week in the early 1970s (MAFF 1974) whereas household purchases had fallen to under half a kilo per person per week in 2023 (Statistica 2024). The consumption of staple foods generally decreases as societies become more prosperous and consume more mixed diets, with a wider range of breads and other baked goods being available than in the past. However, the decreases in wheat consumption also reflect specific concerns about the adverse effects of wheat‐based foods on health. These include the role of highly refined foods (described by some as ultra‐processed foods, (UPFs)) on the risk of developing non‐communicable diseases ((NCDs), obesity, type 2 diabetes, cardiovascular disease) associated with the ‘Western Diet and Lifestyle’ and specific adverse responses to wheat or gluten (notably coeliac disease and non‐coeliac wheat sensitivity, (NCWS)). In the UK, a population study indicated that 3.7% of the population consumed a gluten‐free diet, which exceeds the prevalence of coeliac disease (about 1%) (Croall et al. 2019).

Bread and other staple foods are central to human nutrition and are deeply embedded in the cultures that have consumed them for millennia. It is therefore understandable that consumers are concerned about changes in the types of crops which are grown and the processing systems that are used. This is illustrated by past and current debates about the merits of organic versus conventional production systems, the acceptability of genetically modified and gene edited crops and traditional versus modern types of crops and processes. In the case of bread, there is an active debate about the relative merits of bread made using traditional and modern processes and from modern and older types of wheat. It is therefore necessary to critically evaluate the mixture of facts and myths that surround the current debate on the impacts of different types of bread on health in order to provide evidence‐based advice to food processors, consumers, regulatory authorities and policymakers and to identify knowledge gaps for future research.

This article will therefore question assumptions in order to stimulate informed discussion, directing the reader to more comprehensive accounts where appropriate. It will achieve this by answering a series of questions that underpin the current concerns of consumers and health professionals.

## 2 What Are White, Wholemeal and Wholegrain Breads?

**FIGURE 1:**
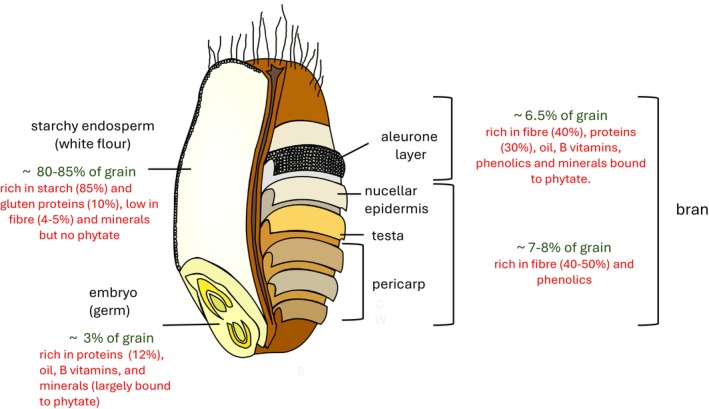
Schematic structure of the wheat grain showing the major tissues in relation to the origins of milling fractions and their compositions. All percentages are on a dry weight basis.

The mature wheat grain is hard and dry and cannot be consumed without processing. Although wheat grains can be consumed after boiling (in the same way as rice), they are usually initially milled to separate the starchy endosperm (the major storage tissue) from the embryo (germ) and the outer layers of the grain and to reduce the particle size of the starchy endosperm to give fine white flour. The outer layers include the micronutrient‐rich aleurone layer (Brouns et al. 2012) (which is the outermost layer of endosperm cells) and together form the bran fraction which, in most milling processes, also contains the germ. Milling therefore has a significant impact on the composition of the flour because many essential and beneficial nutrients are present in higher concentrations in the outer layers and germ compared to the starchy endosperm (see Figure 1). For example, the content of fibre in white flour is about a third of that in the whole grain (about 4%–5% compared with 11%–14% dry weight), while the contents of B vitamins, iron, zinc and phytochemicals (notably phenolic acids which include forms bound to fibre) are also substantially reduced (McCance and Widdowson 2014; Turner et al. 2021).

The starchy endosperm accounts for about 83% of the dry weight of the grain (Barron et al. 2007) and modern roller mills give white flour yields of up to 80% (i.e., about 96% of the theoretical maximum). These yields are achieved by highly sophisticated technologies in which the grain is passed through multiple sets of rollers and sieves to give up to 30 fractions (mill streams) (Miskelly and Suter 2017). The purest of these mill streams are then combined to give white flour. However, it is possible to increase the flour yield by including some bran streams, to give ‘high extraction flours’. This strategy was used in the UK during the Second World War when legislation was introduced to produce ‘National Flour’ with an extraction rate of 85%. Traditional stone milling is less effective at separating the grain tissues and the white flour fractions produced are therefore less pure.

Historically, wholegrain products have been less clearly defined. However, the proposed Healthgrain definition (Ross et al. 2017) is becoming widely accepted and is therefore quoted in full: ‘whole grains shall consist of the intact, ground, cracked, or flaked kernel after the removal of inedible parts, such as the hull and husk. The principal anatomical components—the starchy endosperm, germ, and bran—are present in the same relative proportions as those which exist in the intact kernel. Small losses of components, that is, 2% of the grain or 10% of the bran that occurs through processing methods consistent with safety and quality are allowed’. However, the Healthgrain definition still awaits broad international adoption by food authorities and the definition is still under discussion in the UK. As a result, the legal requirements for labelling products as wholegrain still vary widely between countries, from products containing less than 50% to essentially 100% wholegrains (Ross et al. 2017).

The term wholemeal is widely used in the UK and recognised by consumers, with wholemeal flour defined as comprising the entire edible grain after removal of inedible parts such as the hull and glume and wholemeal breads being made with 100% wholemeal flour (Bread and Flour Regulations 1998). Hence, in the UK wholemeal products really do comprise the whole grain.

Finally, the flours used to make wholegrain and wholemeal breads may vary in their fineness of milling and the size of the particles, from the presence of some cracked or whole kernels to fine homogeneous flours.

In addition to white, wholemeal and wholegrain breads, a range of other types are marketed. These include blends of white and wholemeal flours (sometimes referred to as half and half), blends of wheat and other flours (from other cereals and other seeds), brown flours and flours fortified with fibre (from cereal or other sources) or other components (including various whole or cracked seeds). These types of bread may be more appealing to consumers and are often assumed to have a more favourable nutritional composition than white bread, including higher fibre. However, this may not be the case, particularly for fibre content, which may be assumed to be higher based on the brown colour and texture of the bread but is in fact similar to that in white bread. Consumers should therefore read the labelling of nutrient composition before purchasing!

The differences in composition between types of flours and the variation in their proportions in products therefore pose challenges for interpreting data on health outcomes.

## 3 Do Wholemeal and Wholegrain Breads Have Health Benefits Compared With White Bread?

Regular consumption of whole grain foods is associated with significant reductions in the risks of a range of chronic diseases, including type 2 diabetes, cardiovascular disease and cancer of the colon, and may also have favourable effects on weight management and the composition of the gut microbiota (Ley et al. 2014; Aune et al. 2016; Reynolds et al. 2019; Maki et al. 2019; Gaesser 2020; Wang et al. 2020; Tullio et al. 2020; Guo et al. 2021).

The mechanisms are still incompletely understood, but they appear to be predominantly related to the contents of fibre (derived principally from the bran) and micronutrients that are associated with fibre (Wu et al. 2015; Huang et al. 2015, Zhu and Sang 2017, Barrett et al. 2019, Oh et al. 2019; Reynolds et al. 2019, Veronese et al. 2018, Dahm et al. 2024). In addition, sourdough systems and/or the inclusion of a significant proportion of coarsely milled or intact grains may give a denser bread structure which may reduce the rate of consumption (Heuven et al. 2024) and thus energy intake.

Furthermore, non‐absorbed carbohydrates, fibre and phytochemicals (which include alkylresorcinols, phytosterols, lignans and phenolic acids) may be fermented or converted to other bioactive components by microbiota present in the colon. This can result in favourable effects on the diversity and metabolism of the microbiota, the production of short‐chain fatty acids and the reduction of pH, the softening of stool and reduced transit time, and the reduction of toxic metabolites produced by fermentation of protein, such as ammonia, indoles and *p‐*cresol (Bach Knudsen 2015; Zhu and Sang 2017; Gill et al. 2018, Gill et al. 2021; Tian et al. 2022). It is therefore likely that phytochemicals act ‘in concert’ with fibre to reduce disease risk.

Beneficial effects of phytochemicals, particularly phenolics, in wheat have been shown in short‐term intervention studies, including anti‐inflammatory effects and improved vascular function (Price et al. 2012; Mateo Anson et al. 2011; Turner et al. 2021). This suggests that wholemeal wheat consumption can make an important contribution to overall polyphenol intake, which has been shown to be inversely related to cardiovascular events (Tresserra‐Rimbau et al. 2014; Mendonça et al. 2019).

A commonly held view is that white bread is more rapidly digested than wholemeal and wholegrain, leading to a faster increase in blood glucose and increased risk of type 2 diabetes. However, published studies of wholemeal and white breads show no consistent differences. In particular, Musa‐Veloso et al. (2018) reported a meta‐analysis of eight datasets comparing wholemeal and white bread (six datasets) or pasta (two datasets). The authors state that “when not considering significance (which can be affected by the statistical power of a study), the consumption of bread or pasta composed of wholemeal wheat was associated with a reduction in the blood glucose AUC0–120 min or AUC0–180 min in 3 strata, but with an increase in the blood glucose AUC in 5 strata relative to the consumption of bread or pasta composed of white wheat” and conclude that “overall, the results of the meta‐analysis suggest that the consumption of bread and pasta made with wholemeal wheat, instead of white‐wheat bread and pasta, does not reduce the postprandial blood glucose AUC”. However, they also noted that “the results of 2 studies indicate that the consumption of whole‐grain bread made with intact wheat kernels, instead of white wheat significantly lowers blood glucose AUC”. Hence, greater clarity is required in distinguishing between the precise types of breads used in short‐term intervention studies and monitored in long‐term dietary surveys. Similarly, the meta‐analysis reported by Reynolds et al. (2019) concluded that the evidence for associations between carbohydrate quality and effects on glycaemic index and glycaemic load was low to very low.

While it is clear that high fibre products have benefits in reducing the risk of chronic diseases, and the UK Eatwell Guide recommends that consumers ‘base your meals around starchy carbohydrate foods’ and to ‘choose wholegrain or higher fibre versions’ of foods, fibre intake in the UK still falls far below dietary recommendations, about 12–14g lower than the 30 g a day recommended for UK adults (discussed by Lovegrove et al. 2025). However, the extent to which benefits established for wholegrain foods are shared by wholemeal and other types of ‘nutritionally enhanced breads’ is not clear. Furthermore, white bread still meets the definition of being a source of fibre, as described in the GB nutrition and health claims register, and the recognition that wholegrain products have additional health benefits does not mean that white bread is intrinsically unhealthy. In this respect, it should be noted that the impact of bread on health will depend not only on the amount consumed and its composition but also on other foods consumed with the bread and present in the overall diet. For example, while adding healthy toppings or fillings to white bread will improve its impact on health, adding unhealthy toppings or fillings to whole grain bread will have the opposite effect.

There are also concerns that white bread is more active in triggering adverse reactions to wheat. The grain components of most concern are proteins and FODMAPs (Fermentable Oligo‐, Di‐ and Monosaccharides And Polyols). In particular, the gluten proteins are responsible for triggering detrimental physiological changes in coeliac disease (which currently affects about 1% of the population in the UK (Croall et al. 2019)) while the amylase/trypsin inhibitors (ATIs) have been implicated in ‘non‐coeliac wheat sensitivity’ (Geisslitz et al. 2021). True IgE‐mediated food allergy to wheat has a very low prevalence (a recent report showing 0.25% of Germans aged 12–80 (Neyer et al. 2025)) but a range of proteins have been implicated including gluten proteins and ATIs, with the latter being the major allergens in Bakers' asthma (respiratory allergy) (Geisslitz et al. 2021).

FODMAPs are considered to exacerbate symptoms in irritable bowel syndrome (IBS) (which has an estimated prevalence of 5%–20% in the UK (NICE 2025)) because they are rapidly fermented in the colon leading to the production of gas and an increase in luminal water (Cox et al. 2021; Whelan and Staudacher 2022). The major FODMAPs in wheat grain are fructo‐oligosaccharides (fructans) and raffinose (the trisaccharide galactose glucose fructose) (Ispiryan et al. 2020).

Fructans are more highly concentrated in whole grain than in white flour (Ispiryan et al. 2020). By contrast, gluten proteins are only present (Shewry et al. 2009) and ATIs are enriched (Geisslitz et al. 2021) in the starchy endosperm of the grain and hence in white flour.

## 4 Does the Softer Texture of Ultra‐Processed Breads Affect Intake and Health?

It has been suggested that artisan breads are more favourable for health than ultra‐processed breads since the latter are typically softer and can therefore be consumed more rapidly. Indeed, studies have shown that breads with a thicker crust tend to be ingested more slowly (Gao et al. 2015; Gao and Zhou 2021; Jourdren et al. 2016). However, there is considerable variation across both artisan and factory‐produced breads, and specific products can differ substantially in texture and structure. For example, it has been reported that factory‐produced white bread may be consumed more rapidly than factory‐produced wholemeal bread due to its softer texture (Heuven et al. 2024). Interestingly, sliced bread was consumed more slowly than bread rolls, highlighting the influence of physical form.

Hence, it is clear that food structure (including form and texture) can significantly affect intake, even among foods within the same Nova category (Forde et al. 2020; Lasschuijt et al. 2023; Teo et al. 2022) and ongoing research continues to explore these relationships (Lasschuijt et al. 2025).

However, it should be noted that the fact that white bread may be consumed more rapidly than wholegrain bread does not in itself demonstrate that the product is intrinsically unhealthy or that it leads to increased 24 h energy intake.

## 5 Are Sourdough Breads Healthier Than Yeast‐Fermented Breads?

There is no doubt that sourdough breads differ from yeast‐fermented breads in their flavour, taste and texture. But does sourdough bread, made from white flour or wholegrain, have benefits for health compared with yeast‐fermented bread?

Sourdough fermentation is considered to be a more ancient process than conventional yeast fermentation and probably originated from spontaneous fermentation by microorganisms (bacteria and yeasts) naturally present in grains and flours and in the processing environment. Artisan bakers have their own ‘mother sourdoughs’ which have some common features (notably high levels of lactic acid bacteria and a predominance of bakers' yeast, *Saccharomyces cerevisiae*, in the yeast fraction) but otherwise differ widely in composition with respect to microbiota subclasses and their enzyme and metabolic activities. In fact, over 100 species of lactic acid bacteria, predominantly species of Lactobacillaceae (Zheng et al. 2020), and several dozen yeast species have been isolated from sourdoughs. Consequently, there are many different types of artisan sourdoughs (Landis et al. 2021) which are also processed in different ways by bakers; for example, the proportions of mother dough used and the duration and temperature of fermentation vary widely. In addition to artisan sourdoughs, ingredients companies supply bakers with a variety of tailor‐made sourdough starters, as discussed in detail by De Vuyst et al. (2023), while many bakers also apply a ‘finishing touch’ by adding bakers' yeast to improve bread volume and softness.

The differences in microbial composition and processing conditions therefore result in wide variation in products labelled as ‘sourdough’ and there is no internationally accepted definition or standard of ‘sourdough processing’ (Brandt 2023).

Analyses of breads made using experimental sourdough systems show effects on composition that potentially may be beneficial to health. For example, reduction of components that may induce adverse reactions, such as gluten proteins (Thiele et al. 2014) and ATIs (amylase trypsin inhibitors) (Geisslitz and Scherf 2024), increases in amino acids (notably gamma‐amino butyric acid), ‘bioactive peptides’ and organic acids (lactic acid, acetic acid), and reduced digestibility of starch (Paramithiotis et al. 2024). Sourdough fermentation may also increase the bioaccessibility of minerals (notably iron, zinc and magnesium), by degrading the phytic acid that binds these minerals in the aleurone and germ. Consequently, this effect will be observed in wholemeal and wholegrain breads (Rodriguez‐Ramiro et al. 2017) but not in white bread as white flour should not contain the mineral‐rich aleurone (which is part of the bran), nor phytate (unless from contamination with other fractions).

Both sourdough and yeast processing have also been shown to reduce the content of FODMAPs (fructans and raffinose) present in white and wholegrain flours (Geisslitz and Scherf 2024). This may result in less gas formation in the colon and less intestinal distress, especially in individuals with IBS, and the potential benefits are widely communicated in social media and marketed as health benefits. However, sourdough fermentation also results in the production of mannitol, a fermentable sugar alcohol, and the total FODMAPs content of the dough and bread may actually increase (Shewry et al. 2022).

Furthermore, most of the studies discussed above have been carried out using experimental sourdough systems and have not been demonstrated in products marketed and consumed by the public. These experimental systems often use selected microbiota and/or fungal enzyme fractions (with high protease activity) and/or long fermentation times which result in high dough acidity (a pH below 4.5 compared with above 5 which is common for sourdough breads purchased in Spain (Brandt 2023)). These conditions may result in poor product quality, due to the disruption of the gluten network, and a strongly acidic taste, and therefore low acceptability by consumers.

Compared to yeast‐fermented breads, sourdough wholegrain breads are known to have a denser structure which may reduce the rate of oral processing, consumption and energy intake (Heuven et al. 2024). The inclusion of coarsely ground grains and intact kernels in both yeast and sourdough‐fermented breads may also reduce the rate of digestion and absorption leading to effects on increased satiety and reduced glycaemic response compared to breads made from finely milled grains (Edwards et al. 2015; Aleixandre et al. 2019; Cai et al. 2021; Ying et al. 2024). However, there is no evidence for meaningful clinical effects on satiety, 24‐h energy intake, glycemia and chronic disease risk markers when comparing sourdough breads with yeast breads made from the same ingredients (Ribet et al. 2023; D'Amico et al. 2023).

Consequently, it has been concluded that a sound evidence base for enhanced beneficial effects of sourdough breads on health‐related clinical endpoints compared to yeast‐fermented breads has not been established so far in humans (D'Amico et al. 2023; Ribet et al. 2023) and that the claims that are frequently made for health benefits due to sourdough processing do not generally apply to the sourdough breads that are commonly purchased and consumed.

## 6 Does the Classification of Factory‐Produced Breads as UPFs Mean They Should Be Avoided?

The classification of sliced pre‐packaged breads as ‘ultra‐processed foods’ (UPFs) implies that they have adverse effects on health. This classification is based on the Nova system developed by Monteiro and co‐workers who defined UPFs as ‘formulations of ingredients, mostly of exclusive industrial use, that result from a series of industrial processes (hence “ultra‐processed”)’ (Monteiro et al. 2019). Nova is widely accepted and used in nutrition research, notably in epidemiological studies, and many studies have shown adverse associations of high intakes of foods/drinks classified as UPFs by Nova and a wide range of health outcomes (see, for example, Vitale et al. 2024; Lane et al. 2024).

However, Nova has also been criticised as an oversimplification (Jones 2019; Gibney and Forde 2022; Braesco et al. 2022) as it does not discriminate between individual foods within the broad groups. The complexity of the relationship between UPFs and health is also highlighted by an analysis which showed that although the consumption of UPFs is associated with a deterioration in the overall quality of the diet and positively associated with cardiometabolic risk, this association is mediated by and dependent on the quality of the whole diet rather than just the ultra‐processed component (Griffin et al. 2021). Similarly, Machado et al. (2019) showed that negative effects of UPFs were related to the contents of individual components (notably high free sugars, total, saturated and trans fats, sodium and energy density and low fibre and potassium) present in the foods. Finally, it should be noted that a large multinational cohort study showed that although total intake of UPFs was associated with increased risk of cancer‐cardiometabolic multimorbidity, the consumption of ultra‐processed breads and cereals was associated with reduced risk (Cordova et al. 2023).

Pre‐packaged factory‐produced breads (including white, wholemeal and other breads) made in the UK usually fall into Group 4 (UPF) in the Nova classification, based mainly on the types of ingredients, including emulsifiers, whereas artisan breads typically fall into Nova group 3 (processed foods). We will therefore discuss whether there is scientific evidence that the ingredients used to produce these breads that would be classified as ultra‐processed are intrinsically harmful.

Some of the additives in the factory‐made breads consumed in the UK (as detailed below) relate to the use of the Chorleywood bread process (CBP) (Cauvain and Young 2006), which was introduced in the early 1960s to increase the efficiency and reduce the cost of production. The CBP is a rapid process in which dough mixing and development are carried out in a single operation, using higher energy levels. It requires shorter processing times (and hence has reduced cost) and gives greater product consistency. The CBP allows the use of weaker doughs than conventional breadmaking systems, which may require a lower flour protein content (Cauvain and Young 2006). This may allow the amount of nitrogen fertiliser applied to the crop to be reduced, with economic and environmental benefits. However, it also requires changes to the dough recipe including a higher amount of yeast and the inclusion of additives.

There is a statutory requirement in the UK to state the ingredients on food packaging and comparisons of pre‐packaged sliced breads (both white and wholemeal) marketed in the UK show similar additives. These include natural ingredients: soya flour, rapeseed oil and ascorbic acid (vitamin C). They also include calcium propionate (E282) as a preservative; this is the calcium salt of propionic acid which occurs naturally, particularly in dairy products. It has been used and regarded as safe for over 50 years and propionate is in fact one of the ‘beneficial’ short‐chain fatty acids produced by the fermentation of fibre by bacteria in the colon. CBP bread also contains added fat and/or an emulsifier which may be mono‐ and diglycerides (E471) (which occur naturally in plants and are released by digestion of triglycerides), mono‐ and diacetyl tartaric esters of mono‐ and diglycerides (E472e, also called DATEM) and/or sodium stearoyl‐2‐lactylate (E481). Enzymes may also be used as processing aids that speed up favourable biochemical reactions, improving the texture, taste and overall quality of the bread, notably amylase to partially digest starch to provide free glucose to support the fermentation process. Finally, white breads are frequently fortified with essential minerals and B vitamins (as discussed below). Most of the additives in pre‐packaged factory‐made breads are therefore either present naturally in foods or based on naturally occurring molecules and all additives used in the UK and EU have undergone extensive testing to establish their safety before approval for food use (EFSA NDA Panel 2024; FSA 2025).

Emulsifiers are widely used in processed foods, with a survey of over 32 000 products in the UK showing that over half contained at least one emulsifier (Sandall et al. 2023). However, in recent years concerns have been raised about the effects of emulsifiers on health, and it has been suggested that they may contribute to the pathogenesis of inflammatory bowel disease (IBD) (Bancil et al. 2021). In response, studies have been initiated to determine whether restricting emulsifier intake could be beneficial for patients with gut‐related conditions including Crohn's disease (Bancil et al. 2021, 2025). It is clearly important that any potential adverse effects of food additives are thoroughly investigated and their use re‐evaluated when new evidence emerges. In the case of emulsifiers, a recent review concluded that further research is needed to clarify their role in IBD and to determine whether any observed effects are relevant to the health of the general population (Bancil et al. 2025).

## 7 What Is the Real Contribution of Factory‐Produced White Bread to Nutrition and Health?

The benefits of wholegrain have been promoted for over 30 years. The success of this campaign cannot be accurately quantified as we do not know what the pattern of consumption would have been without the promotion. However, we do know that the proportion of wholemeal and brown breadmaking flours produced by UK millers is actually decreasing, from about 14.9% of the total in 2011–12 to 9% in 2022–2023 (Shewry et al. 2023). By contrast, national whole grain promotion campaigns in Denmark may have contributed to a significant increase in whole grain food consumption in recent years, with average wholegrain intake of Danish adults increasing from 33 g/day in 2000–2004 to 55 g/day in 2011–2012 (Mejborn et al. 2013). This represents a formidable effort and is a good example of a successful initiative to improve public health (Boyle et al. 2024). However, such increases in the consumption of wholegrain products are rare and white bread remains dominant in much of the world, particularly in countries where bread consumption is high (Turkey and the Balkans, Middle East, North Africa and Central Asia). In the UK, white bread represents 43% of the wheat bread consumed among adults aged 19–64 years, with smaller quantities of wholemeal and mixed grain wheat bread being consumed, while 63% of adults are consumers of white bread (OHID 2025).

**FIGURE 2:**
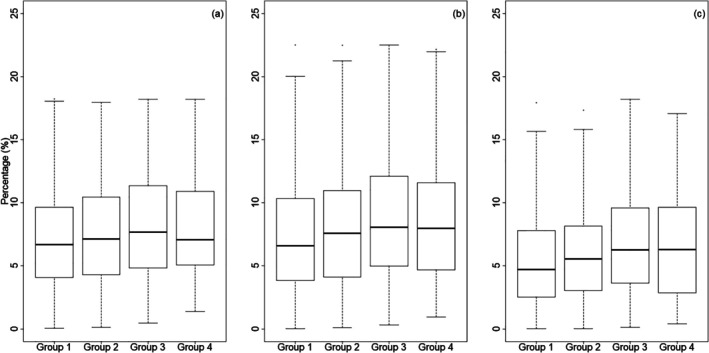
Percentage contributions of white bread to dietary intakes of (a) energy, (b) fibre and (c) folate in UK adults calculated from the UK National Diet and Nutrition Survey, rounds 9–11. Participants are grouped by occupation, using Office of National Statistics categories: Group 1, Higher managerial and professional occupations and Lower managerial and professional occupations; Group 2, Intermediate occupations and small employers and own account workers; Group 3, Lower supervisory and technical occupations, Semi‐routine occupations and Routine occupations; Group 4, Never worked and Long‐term unemployed. Boxes display median and interquartile range (IQR); the lower whisker is the minimum of the range and the upper whisker is the third quartile +1.5*IQR.

Consumers value the affordability, convenience (including shelf life) and palatability of white bread over wholemeal (Lockyer and Spiro 2020; Norton et al. 2024). Furthermore, despite losses on milling, white bread still contributes substantial proportions of energy, macronutrients and micronutrients to the UK diet as it is widely consumed across socio‐economic groups. For example, through analysis of individual‐level dietary data from the UK National Diet and Nutrition Survey (NDNS; rounds 9–11, years 2016–2019) (University of Cambridge, MRC Epidemiology Unit 2023), we calculate that white bread contributes approximately 7% (IQR: 4%–11%), 7% (IQR: 4%–12%) and 5% (IQR: 3%–9%) of total dietary energy, fibre and folate, respectively, among adults in the UK, with greater contributions among those employed in semi‐routine and routine occupations compared to those in managerial occupations (Figure 2).

## 8 Why Is White Bread Fortified?

The depletion in mineral micronutrients and B vitamins by milling, and the importance of bread as a source of these, is recognised in many countries by mandatory fortification of white breadmaking flour, to increase the levels up to or above those present in wholemeal flour (Bread and Flour Regulations 1998). For example, white breadmaking flour is fortified with iron and calcium in the UK, with white bread accounting for 7%–10% and 8%–12% of the intakes of these minerals, and wholemeal bread contributing 2%–5% and 1%–3% respectively, across age groups (Lockyer and Spiro 2020). Modelling conducted by the UK Scientific Advisory Committee on Nutrition concluded that removal of this fortification would have significant negative effects on the proportions of young people aged 11–18 years and females aged 19–64 years reaching the Lower Reference Nutrient Intake (LRNI) for calcium and of older girls and women for iron (SACN 2012). By contrast, although white flour in the UK is currently fortified with thiamin (vitamin B1) and niacin (vitamin B3), modelling showed that removal of this fortification would have little impact on the intakes of these vitamins (SACN 2012).

By the end of 2026, commercial millers in the UK will be required to fortify white flour with folic acid, the synthetic form of folate (vitamin B9), in order to reduce the incidence of neural tube defects in babies with no requirement for consumers to change their eating behaviour. White bread currently contains an average of 29 μg/100 g folates compared to 40 μg/100 g in wholemeal bread (McCance and Widdowson 2014). The level of fortification with folic acid is 250 μg/100 g flour which equates to about 150 μg per 100 g bread. Fortification will therefore have a substantial effect on folate intake from white bread, with lower income groups likely to incur the greatest benefits due to their lower baseline folate intakes and the larger contribution of white bread to their total dietary folate intakes (Figure 2). Small‐scale millers (producing less than 500 metric tonnes of flour per year) are exempt from these regulations, as are wholemeal breads.

## 9 Conclusions

Breads made from wholemeal and wholegrain flours and using traditional processes (either sourdough or yeast‐fermented) are often marketed on the basis of health benefits and consumers are also led to believe that traditional foods without ‘additives’ are healthier. We have therefore briefly discussed the scientific evidence for these contentions.

Firstly, there is no doubt that wholemeal and wholegrain breads are beneficial in contributing higher amounts of dietary fibre (and associated micronutrients and phytochemicals), although the relationship between the particle size of flours (i.e., wholemeal compared with wholegrain flours) and behaviour in the GI tract is still incompletely understood. In addition, further research on the impact of bread texture on consumption and health outcomes is warranted. Phytochemicals, which are concentrated in the bran, may also have health benefits but these have not been approved for health claims. The depletion of B vitamins and minerals in white flour is not a concern in many countries, including the UK, as fortification is carried out. Furthermore, the low intrinsic levels of minerals in white flour may actually be more bioavailable than the higher levels of minerals in wholemeal due to the absence of phytic acid (Eagling et al. 2014).

Similarly, scientific comparisons have so far failed to show significant differences between the effects of traditional and modern breadmaking processes on the quality of bread for human health, and suggestions of adverse effects of the additives and improvers which are widely used in factory bread production need to be substantiated. Our conclusion is consistent with the recent statement on processed foods and health from the UK Scientific Advisory Committee on Nutrition which concluded that there is a risk that over‐reliance on UPF categories in public health messaging may mislead consumers (SACN 2023).

While the consumption of wholegrain should continue to be encouraged, white bread should not be regarded as inherently unhealthy. Furthermore, the promotion of artisan products should target personal taste rather than health benefits until scientific evidence is produced to substantiate these claims. This is important because breads made using artisanal processes are more expensive than modern factory‐made bread. The ability to produce bread with good nutritional quality and at much lower cost means that factory‐made bread, and particularly white bread, will remain an important source of energy and nutrients in the UK and global diets. This is particularly true for low‐income households, which spend a higher proportion of their budget on food, and an increase in the costs of bread has the potential to widen economic and health inequities. Thus, rather than discouraging the consumption of white bread, it should be recognised that it already contributes significantly to nutrition and health and that further improvement of its nutritional value, through crop improvement, processing or fortification, can offer effective and equitable ways to improve human health outcomes.

## Author Contributions

Conceptualisation: P.R.S., A.L., E.J.M.J., G.F., F.B.; writing – original draft: P.R.S., A.L., E.J.M.J. G.F., F.B.; writing – review and editing: P.R.S., A.L., E.J.M.J. G.F., F.B.; supervision: E.J.M.J.; formal analysis: L.S.R.

## Funding

This work was supported by Biotechnology and Biological Sciences Research Council, BB/X010953/1, BB/X011003/1, BBS/E/RH/230003C.

## Conflicts of Interest

The authors declare no conflicts of interest.

## Data Availability

Data used to prepare Figure 2 and to calculate values quoted in the text are available from the authors. No other new data were used.
